# New Insights into the Toxin Diversity and Antimicrobial Activity of the “Fire Coral” *Millepora complanata*

**DOI:** 10.3390/toxins14030206

**Published:** 2022-03-14

**Authors:** Víctor Hugo Hernández-Elizárraga, Andrea Ocharán-Mercado, Norma Olguín-López, Rosalina Hernández-Matehuala, Juan Caballero-Pérez, César Ibarra-Alvarado, Alejandra Rojas-Molina

**Affiliations:** 1 Posgrado en Ciencias Químico Biológicas, Facultad de Química, Universidad Autónoma de Querétaro, Santiago de Querétaro 76000, Mexico; vhelizarraga@gmail.com (V.H.H.-E.); normita_39@hotmail.com (N.O.-L.); 2 Laboratorio de Investigación Química y Farmacológica de Productos Naturales, Facultad de Química, Universidad Autónoma de Querétaro, Santiago de Querétaro 76000, Mexico; anmayener@hotmail.com (A.O.-M.); rosalinahm1@yahoo.com.mx (R.H.-M.); cibarra@uaq.mx (C.I.-A.); 3 European Molecular Biology Laboratory’s European Bioinformatics Institute, Wellcome Genome Campus, Hinxton CB10 1SD, UK; jcaballero@ebi.ac.uk

**Keywords:** *Millepora complanata*, transcriptomics, toxin diversity, cytolysins, antibacterial peptides

## Abstract

To date, few studies have been carried out aimed at characterizing the toxins synthesized by hydrocorals of the genus *Millepora*. The purpose of this study was to explore the toxin diversity and antibacterial activity of the “fire coral” *M. complanata* using a transcriptomic data mining approach. In addition, the cytolytic and antibacterial activities of the *M. complanata* nematocyst proteome were experimentally confirmed. Cytolysins were predicted from the transcriptome by comparing against the Animal Toxin Annotation Project database, resulting in 190 putative toxins, including metalloproteases, hemostasis-impairing toxins, phospholipases, among others. The *M. complanata* nematocyst proteome was analyzed by 1D and 2D electrophoresis and zymography. The zymograms showed different zones of cytolytic activity: two zones of hemolysis at ~25 and ~205 kDa, two regions corresponding to phospholipase A2 (PLA2) activity around 6 and 25 kDa, and a proteolytic zone was observed between 50 and 205 kDa. The hemolytic activity of the proteome was inhibited in the presence of PLA2 and proteases inhibitors, suggesting that PLA2s, trypsin, chymotrypsin, serine-proteases, and matrix metalloproteases are responsible for the hemolysis. On the other hand, antimicrobial peptide sequences were retrieved from their transcripts with the amPEPpy software. This analysis revealed the presence of homologs to SK84, cgUbiquitin, Ubiquicidin, TroTbeta4, SPINK9-v1, and Histone-related antimicrobials in the transcriptome of this cnidarian. Finally, by employing disk diffusion and microdilution assays, we found that the nematocyst peptidome of *M. complanata* showed inhibitory activity against both Gram-positive and Gram-negative bacteria including *S. enteritidis*, *P. perfectomarina*, *E. coli*, and *C. xerosis*, among others. This is the first transcriptomic data mining analysis to explore the diversity of the toxins synthesized by an organism of the genus *Millepora*. Undoubtedly, this work provides information that will broaden our general understanding of the structural richness of cnidarian toxins.

## 1. Introduction

Cnidarians are simple and ancestral organisms considered as the oldest lineage of poisonous animals, which have the ability to synthesize molecules capable of inducing different toxic effects and lethality [1]. Cnidarian venoms are stored in the nematocysts, specialized organelles for defense and prey capture [2]. These complex chemical mixtures contain mainly peptidic and proteinaceous components, which have been categorized into three principal groups, based on their mechanism of action: (a) enzymes (e.g., metalloproteases and phospholipases A2 (PLA2s)) [1,3]; (b) pore-forming toxins (PFTs) (e.g., actinoporins, jellyfish toxins (JFTs), hydralysins (Hlns), etc.) [4]; and (c) neurotoxins (e.g., toxins that target voltage-gated sodium channels (NaTXs), toxins targeting voltage-gated potassium channels (KTXs), Kunitz peptides, etc.) [5]. PLA2 and metalloprotease activities have been widely described for cnidarian venoms [6]. Particularly, PLA2s have been found in cnidarians of the classes Anthozoa, Scyphozoa, Cubozoa, and Hydrozoa [4].

Cnidarian toxins possess a wide spectrum of biological effects, for instance, PLA2s hydrolyze the *sn-2* ester bond of glycerophospholipids producing lysophospholipids and fatty acids [7]. These toxins also display other pharmacological effects, such as neurotoxicity [8]. Regarding cnidarian venom metalloproteinases, which are metal-dependent peptidases, they are responsible for several toxic effects, including skin damage, myonecrosis, edema, and inflammation [6]. On the other hand, PFTs play an important role in cnidarians’ defense. The cytolysis induced by PFTs involves the formation of transmembrane pores, which leads to cellular disruption due to osmotic imbalance [4,9,10]. The most-studied group of cnidarian PFTs are actinoporins (e.g., equinatoxin II). Cnidarians can also synthesize neuroactive toxins [5], which are employed for immobilizing preys and predators by modulating gating of voltage-dependent sodium channels or blocking voltage-dependent potassium channels during the repolarization stage [11].

In addition to toxins, cnidarians synthesize other types of bioactive molecules, which represent valuable potential sources of lead compounds useful for the development of new drugs [12]. Some of these compounds have antibiotic activity and constitute an important part of the innate immune response of cnidarians, which inhabit hostile environments abundant in bacteria, viruses, and parasites [13].Thus, these marine organisms use their toxins and antimicrobial compounds to face intra and interspecific competition and to defend themselves against microbial pathogens [14]. In fact, a significant antimicrobial activity against a broad spectrum of Gram-positive and Gram-negative bacteria has been demonstrated for numerous cnidarian extracts [15]. Interestingly, several peptides from cnidarians, mainly from the Hydrozoa class, have shown significant antimicrobial activity against multi-resistant strains of Gram-positive and -negative bacteria (e.g., Hydramacin-1, Periculin-1, Kazal-2, and Arminin) [13].

Hydrocorals of the genus *Millepora* are very important from an ecological perspective, as they are important builders of coral reefs. These cnidarians, commonly known as “fire corals”, are also toxinologically relevant, since when coming into contact with human skin, they induce local and systemic effects that include severe pain, blisters, eruptions, nephrotic syndrome, pulmonary edema, and acute renal failure [16]. To date, few studies have been carried out on the toxins produced by organisms of the genus *Millepora*, compared to the wide range of toxinological investigations that have been carried out on other cnidarians. These studies have revealed that “fire corals” produce similar toxic effects. It has been reported that the venoms of *M. dichotoma* and *M. platyphylla* caused violent seizures and death in less than a minute when administered intravenously in mice, in addition to inducing hemolysis and dermonecrosis [17,18]. Similarly, *M. alcicornis* and *M. tenera* elicited lethal effects, hemolysis, and dermonecrosis in mice [19,20,21].

Previous studies carried out by our research group demonstrated that the aqueous extract of *M. complanata* elicited calcium-dependent contractions in guinea pig ileum and rat aorta [22]. Moreover, intravenous administration of this extract caused severe seizures and immediate death in mice with a lethal dose 50 (LD50) of 4.62 µg protein/g of body weight. Doses lower than the LD50 induced lung and kidney injury ascribed to the presence of cytolysins [23]. Interestingly, we found evidence that the aqueous extract of *M. complanata* contains heat-stable toxins of non-protein nature, capable of inducing convulsions and lethality in mice [23].

Our group has also been working with another *Millepora* species, *M. alcicornis*. We found that the aqueous extract of this hydrocoral caused hemolysis of rat erythrocytes, displayed PLA2 activity, and induced a concentration-dependent contraction of rat aortic rings. When intravenously administered, the extract was lethal to mice (LD50 of 17 μg protein/g of body weight), and provoked kidney, liver, and lung damage [24]. A direct zymography analysis showed that this extract contained two types of hemolysins; some of them were proteins with molecular mass ranging from 28 to 30 kDa, which possessed PLA2 activity, and the others had a molecular mass of approximately 200 kDa and did not elicit PLA2 activity [24].

Despite their toxinological importance, there is very little information regarding the primary structure of hydrocoral toxins. The amino acid sequence of only one of them has been determined, an 18 kDa protein (MCTx-1) purified from *M. dichotoma*, whose primary structure was deduced from its cDNA. This toxin showed homology with dermatopontins, which are proteins of the extracellular matrix of mammals [25]. Recently, by using a proteomics approach, we identified five toxin-like sequences in the soluble proteome of *M. complanata* corresponding to two PLA2s (acidic PLA2 PA4 and acidic calcium-independent PLA2-like protein), two PFTs (echotoxin-2 and DELTA-actitoxin-Oor1b), and one metalloprotease (Predicted: astacin-like metalloprotease toxin 5) [26]. In addition, we detected putative toxin sequences, including a metalloprotease (Disintegrin and metalloproteinase domain-containing protein 7), a PLA2, and an actitoxin (DELTA-actitoxin-Ate1a like) in the soluble proteome of *M. alcicornis* [27].

Although the lethality and cytolytic activity of *Millepora* crude extracts have been previously described, very few efforts have been addressed in order to characterize the toxins synthesized by these species; at present, their chemical structure and their mechanisms of action have not been fully described. The aim of the present study was to explore the diversity of *M. complanata* toxins and to predict the presence of antimicrobial peptides using a transcriptome data mining approach. In addition, the cytolytic and antibacterial activities of the proteome of *M. complanata* nematocysts were experimentally confirmed.

## 2. Results

### 2.1. Annotation and GO Terms Assignment

**Figure 1:**
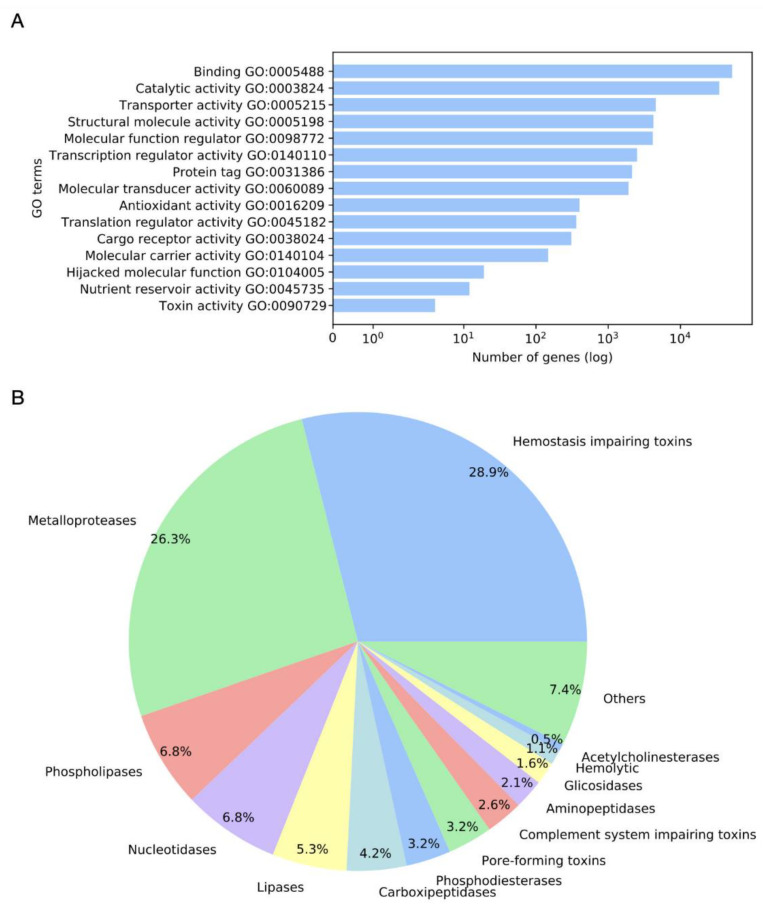
(**A**) Overrepresented gene ontology (GO) terms across the molecular function (MF) subontology and (**B**) toxin distribution from the *M. complanata* transcriptome.

Overall, 412,660 sequences from the *M. complanata* transcriptome were analyzed (DDBJ/EMBL/GenBank accession GIXC00000000). Annotation by sequence homology against the UniProtKB/Swiss-Prot database resulted in 75,287 hits. The mapping analysis showed ontology candidates for 74,949 sequences. Gene ontology (GO) terms were assigned to 66,788 sequences. Overrepresented GO terms across the molecular function (MF) subontology are displayed in Figure 1A. Binding (GO0005488), catalytic activity (GO0003824), transporter activity (GO0005215), structural molecule activity (GO0005198), molecular function regulator (GO0098772), and transcription regulator activity (GO0140110), among others, were highly represented terms in *M. complanata* transcriptome.

### 2.2. Detected Putative Toxins

**Figure 2:**
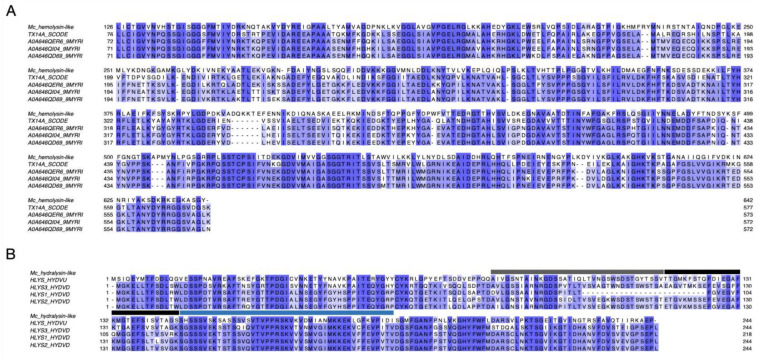
(**A**) Multiple sequence alignment between a hemolysin-like protein from *M. complanata* (Mc_hemolisyn-like) and four centipede toxins (TX14A_SCODE or Scoloptoxin SSD14, A0A646QER6_9MYRI, A0A646QI04_9MYRI, and A0A646QD69_9MYRI) (Amino acid conservation is highlighted in blue). (**B**) Multiple sequence alignment between a *M. complanata* hydralysin-like (Mc_hydralysin-like) toxin with four hydralysins (HLYS_HYDVU, HLYS1_HYDVU, HLYS2_HYDVU, and HLYS3_HYDVU). Annotations: Upper flanking motif between positions 80–115 (gray line); region responsible for transmembrane pore formation at positions 116–145 (black line); and lower franking motif within positions 146–190 (blue line). (Amino acid conservation is highlighted in blue).

The *M. complanata* predicted proteome resulted in 2,475,960 hypothetical proteins. Annotation against the Animal Toxins Annotation Project database showed 3024 hits. After filtering, overall, 190 putative toxins were detected. According to their molecular function, toxins were classified as follows: metalloproteases, hemostasis-impairing toxins, phospholipases, nucleotidases, lipases, carboxypeptidases, phosphodiesterases, PFTs, complement system-impairing toxins, aminopeptidases, glycosidases, acetylcholinesterases, and others. The distribution of identified putative toxins is displayed in Figure 1B. Several *M. complanata* putative toxins showed high sequence similarity with previously reported toxins, two representative alignments of a hemolysin-like toxin and a hydralysin-like toxin are shown in Figure 2.

### 2.3. Virtual Screening of Antimicrobial Peptides (AMPs)

Overall, 1,459,793 protein sequences with length smaller than 100 amino acids were analyzed by amPEPpy. After prediction of antimicrobial peptides (AMPs), 1,397,261 sequences displayed at least one physicochemical-like property related to antimicrobial activity. Derived from annotation against AMPs databases, 1966 hits were obtained from the APD3 database, whereas 3875 hits were recovered from the DRAMP database. Most of the hits showed sequence similarity with SK84, cgUbiquitin, Ubiquicidin, TroTbeta4, SPINK9-v1, and Histone-related antimicrobials. The top ten AMP hits are shown in Table 1.

### 2.4. Electrophoretic Analysis of the M. complanata Nematocysts Proteome

**Figure 3:**
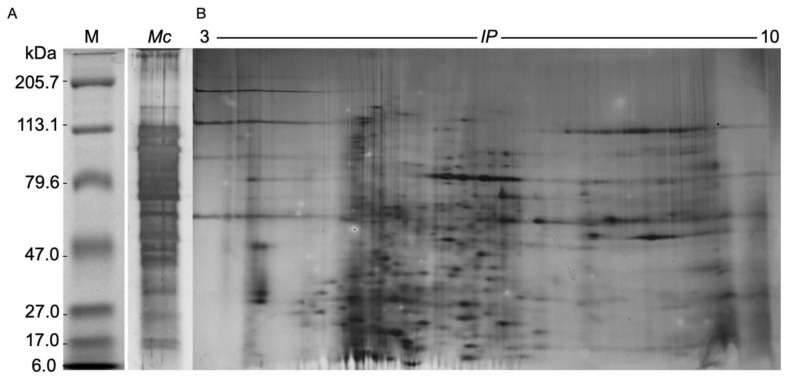
(**A**) SDS-PAGE electrophoretic profile (*Mc*), and (**B**) Two-dimensional electrophoretic profile of the nematocyst proteome of *M. complanata.* MW—molecular mass (kDa). IP—isoelectric point (pH).

Approximately 200 g of *M. complanata* fragments were employed for obtaining the nematocyst proteome. After protein extraction, 1.0 g of lyophilized powder was obtained (yield 0.5%). The total protein content of the lyophilized proteome was 50 ± 13 µg of protein per mg. The SDS-PAGE electrophoretic analysis showed that the nematocyst proteome of *M. complanata* contained peptides and proteins with molecular mass ranging from 6 to 200 kDa (Figure 3A). The 2DE analysis allowed the detection of 575 protein spots, which showed isoelectric point (IP) values between 3 to 10 (Figure 3B).

### 2.5. Zymography Assay

**Figure 4:**
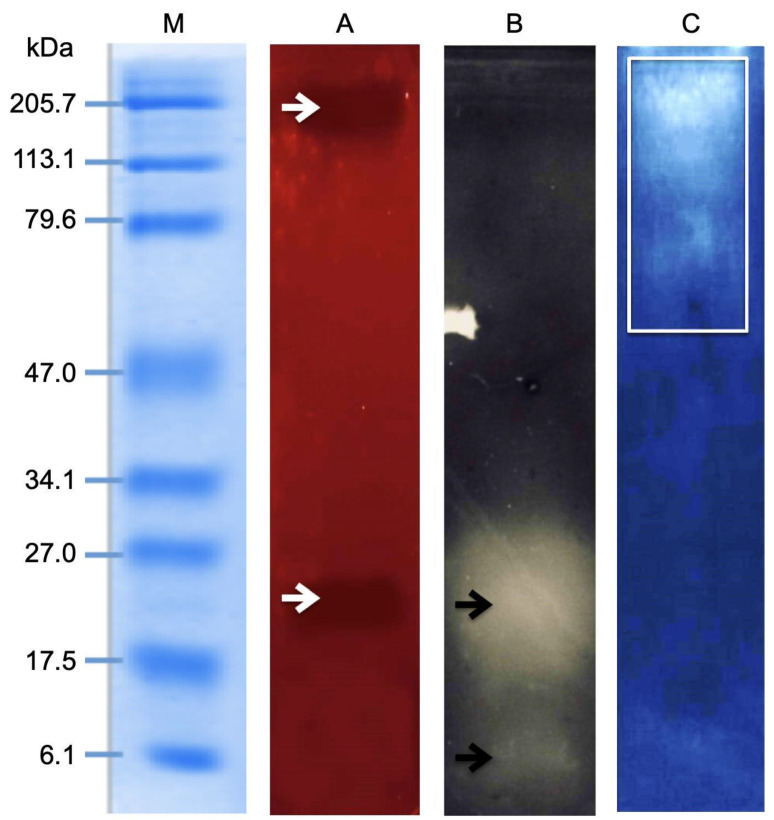
Zymographic analysis. (**A**) Hemolytic activity, two zones of hemolysis at ~25 and ~205 kDa. (**B**) Regions corresponding to PLA2 activity around 6 and 25 kDa. (**C**) Proteolytic activity observed within a molecular mass range of 50 to 205 kDa.

The results from the zymography analysis are displayed in Figure 4. Two zones of hemolysis were detected at ~25 and ~205 kDa (Figure 4A). Zymography assays of PLA2 showed the presence of two protein bands around 6 and 25 kDa (Figure 4B), whereas proteolytic activity was observed within a molecular mass range of 50 to 205 kDa (Figure 4C).

### 2.6. Effect of Enzymatic Inhibitors on the Hemolytic Activity of the M. complanata Nematocysts Proteome

**Figure 5:**
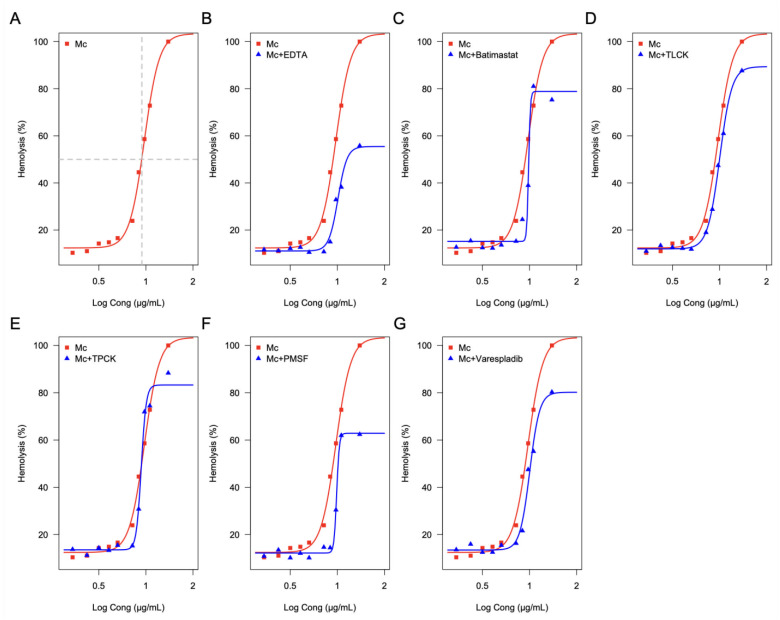
Percentage of the inhibitory effect of enzymatic inhibitors on the hemolytic activity of the proteome of *M. complanata* nematocysts. (**A**) Hemolytic activity (control), (**B**) 44.2% for EDTA, (**C**) 19.68% for Batimastat, (**D**) 2.36% for TLCK, (**E**) 11.59% for TPCK, (**F**) 37.59% for PMSF, and (**G**) Varespladib with 19.66% inhibition.

The proteome of *M. complanata* nematocysts elicited a concentration-dependent hemolysis of human erythrocytes with a hemolytic unit 50 (HU50) value of 9.49 ± 1.03 μg of protein per mL (Figure 5A). All six enzymatic inhibitors diminished the maximal hemolytic activity of the proteome. Inhibition of the hydrocoral nematocysts proteome-mediated hemolysis induced by each inhibitor was: 44.2% for Ethylenediaminetetraacetic acid (EDTA, non-selective protease inhibitor) (Figure 5B), 19.68% for BB-94 (2R,3S)-N4-Hydroxy-N1-[(1S)-2-(methylamino)-2-oxo-1-(phenylmethyl)ethyl]-2-(2-methylpropyl)-3-[(2-thienylthio)methyl] butanediamine (Batimastat, matrix metalloprotease inhibitor) (Figure 5C), 12.36% for Nα-Tosyl-L-lysine chloromethyl ketone hydrochloride (TLCK, trypsin inhibitor) (Figure 5D), 11.59% for N-p-Tosyl-L-phenylalanine chloromethyl ketone (TPCK, chymotrypsin irreversible inhibitor) (Figure 5E), 37.59% for Phenylmethylsulfonyl fluoride (PMSF, serine protease inhibitor) (Figure 5F), and 19.66% for 2-[[3-(2-Amino-2-oxoacetyl)-2-ethyl-1-(phenylmethyl)-1H-indol-4-yl]oxy]-acetic acid (Veraspladib, sPLA2 inhibitor) (Figure 5G).

### 2.7. Antibacterial Activity Induced by M. complanata Nematocysts Peptidome

The susceptibility of bacterial strains to the effect of the *M. complanata* nematocysts peptidome is shown in Table 2. These results showed that this fraction of the nematocyst proteome has antibacterial effects against seven Gram-negative and three Gram-positive bacteria. In the case of Gram-negative bacteria, the largest inhibition zone was 22 ± 1 mm corresponding to *Escherichia coli*, whereas, in the case of Gram-positive bacteria, the largest inhibition zone diameter (20 ± 0.5 mm) was observed for *Bacillus koreensis*.

The antibacterial microdilution method indicated that *Salmonella enteritidis, Pseudomonas perfectomarina, Escherichia coli,* and *Corynebacterium xerosis* were the most sensitive strains with minimum inhibitory concentration (MIC) values of 0.004 μg of protein per mL.

## 3. Discussion

### 3.1. Diversity of M. complanata Toxins and Prediction of Antimicrobial Peptides

Organisms of the Phylum Cnidaria are respected as the most venomous marine organisms on Earth, whose toxins production is crucial for their survival [28]. Here, we explored the toxin diversity synthesized by the “fire coral” *M. complanata* and predicted the presence of antimicrobial peptides by using a transcriptome data mining approach. After transcriptome annotation and GO analysis, several terms were found across the MF subontology. However, “toxin activity” was within the most overrepresented terms (Figure 1A) and 190 sequences from the transcriptome resembled previously reported toxins. These results support the diversity of toxins synthesized by cnidarians of the genus *Millepora* [16,22,23,26].

In this study, the putative toxins detected in the *M. complanata* nematocysts proteome included: metalloproteases, hemostasis-impairing toxins, phospholipases, nucleotidases, lipases, carboxypeptidases, phosphodiesterases, pore-forming toxins, complement system-impairing toxins, aminopeptidases, glycosidases, and acetylcholinesterases, among others (Figure 1B). However, metalloproteases, hemostasis-impairing toxins, and phospholipases (comprising phospholipases A and B) were predominant. Although hemolytic and phospholipase A2 activities have been previously described for some *Millepora* species [18,29,30], the presence of sequences associated with nucleotidases, lipases, carboxypeptidases, phosphodiesterases, PFTs, complement system-impairing toxins, aminopeptidases, glycosidases, and acetylcholinesterases was evidenced for the first time in an organism of the genus *Millepora*.

Our results showed that more than fifty percent of the putative detected toxins corresponded to metalloproteases and hemostasis-impairing toxins. A previous study, carried out on the tentacle transcriptome of the jellyfish *Chrysaora fuscescens*, revealed highly expressed genes encoding metalloproteases, which have a key role during envenomation and cause inflammation and tissue disruption [31]. Further research study performed on the transcriptome of the jellyfish *Cyanea capillata* indicated the high abundance of transcripts encoding metalloproteases [32]. Our study indicates that metalloproteases are important components of hydrocoral venoms, similar to what happens with other cnidarians such as jellyfishes.

On the other hand, hemostatic and hemorrhagic toxins are common components of the venoms of crustaceans, blood feeding insects, leeches, snakes, and some cnidarians. For example, it was found that in the transcriptome of four cerianthid species (*Pachyceriantus* cf. *maua, Isarachnanthus nocturnus, Ceriantheomorphe brasiliensis,* and *Pachycerianthus borealis*), more than 30% of transcripts related to toxin synthesis had homology to transcripts encoding hemostatic or hemorrhagic proteins [33]. The results obtained from the transcriptome analysis of *M. complanata* nematocysts proteome indicate that hemostasis-impairing toxins play an important role in the mechanisms of toxicity induced by the “fire corals” for prey hunting and defense against predators.

One of the main biological effects demonstrated for extracts and venoms obtained from *Millepora* species is the hemolysis that they produce in erythrocytes from different species. However, the chemical structure of hemolysins synthesized by “fire corals” has not been characterized. A putative hemolysin and a putative PFT identified from the *M. complanata* transcriptome were selected for multiple sequence alignment analysis in order to identify conserved structural motifs that are present in PFTs and hemolysins previously identified in hydrozoans and in animals that do not belong to the phylum Cnidaria (Figure 2). Amino acid conservation (highlighted in blue) between a hemolysin-like protein (642 amino acids length) from *M. complanata* (Mc_hemolysin-like) and four centipede toxins was observed: one from a Thai centipede *Scolopendra dehaani* hemolysin (TX14A_SCODE or Scoloptoxin SSD14) and three from *Hemiscolopendra marginata* (A0A646QER6_9MYRI, A0A646QI04_9MYRI, A0A646QD69_9MYRI) (Figure 2A). It was previously found that Scoloptoxin SSD14 elicited a dose-dependent human platelet aggregation (maximum response at 3.2 µg/mL) and induced hemolysis on mice and rabbit erythrocytes (35 and 65% at 5 µg/mL, respectively) [34]. In addition, a hydralysin-like toxin (244 amino acids length) was identified from the *M. complanata* transcriptome. This protein sequence was similar to that of hydralysins from the green hydra *Chlorohydra viridissima*. Hydralysins and their homologs share a group of conserved motifs with known pore-forming toxins such as aerolysins [35]. The *M. complanata* hydralysin-like toxin (Mc_hydralysin-like toxin) detected in this work showed conserved sequence motifs of four hydralysins (HLYS_HYDVU, HLYS1_HYDVU, HLYS2_HYDVU, and HLYS3_HYDVU) (Figure 2B). Specifically, three characteristic conserved motifs (highlighted in blue) from aerolysins were identified in the Mc_hydralysin-like toxin: a) the upper flanking motif between positions 80–115 (gray line); b) the region responsible for transmembrane pore formation at positions 116–145 (black line); and c) the lower franking motif within positions 146–190 (blue line) (Figure 2B) [35]. These results suggest that PFTs, such as hydralysins, may be synthesized by *M. complanata* and they could display mechanisms of pore formation similar to aerolysins. Moreover, it is very likely that proteins structurally related to these types of toxins contribute to the hemolysis induced by *Millepora* venoms.

On the other hand, it has been recognized that cnidarians are valuable sources of antimicrobial peptides [13]. Therefore, a virtual screening for identifying antibacterial peptides in the peptidome of *M. complanata* nematocysts was carried out. Most of the hits from the predicted peptidome showed sequence similarity to SK84, cgUbiquitin, Ubiquicidin, TroTbeta4, SPINK9-v1, and Histone-related antimicrobials (Table 1). After the bioinformatics analysis, the antimicrobial activity of the *M. complanata* nematocysts peptidome against Gram-negative and Gram-positive bacteria was confirmed using the diffusion method. The results demonstrated that, in fact, the hydrocoral peptidome possessed broad-spectrum antibacterial activity against both Gram-negative and Gram-positive groups, with *S. enteritidis, P. perfectomarina, E. coli,* and *C. xerosis* being the most sensitive strains (Table 2). One of the AMPs identified in this work corresponds to cgUbiquitin. According to a previous study, the AMP cgUbiquitin, found in the Pacific oyster *Crassostrea gigas,* possesses potent antibacterial activity against Gram-positive and -negative bacteria. Particularly, this non-hemolytic AMP elicits an antimicrobial effect (through a non-lytic mechanism) on *Streptococcus iniae* and *Vibrio parahaemolyticus* with a minimal effective concentration of 7.8 and 9.8 μg/mL, respectively [36]. Additionally, homologs to SPINK9-v1 were found in the transcriptome of *M. complanata*. SPINK9 and their variants are epidermal antimicrobial peptides synthesized by human skin that selectively kill *E. coli* by a mechanism that involves cell membrane and cytoplasm targets [37]. Ubiquicidin was another potential AMP detected in the *M. complanata* transcriptome. This peptide displayed significant antimicrobial activity against *Listeria monocytogenes*, *Salmonella typhimurium, Escherichia coli, Staphylococcus aureus,* and an avirulent strain of *Yersinia enterocolitica.* Ubiquicidin restricts the intracellular growth of microorganisms in the cytosol of macrophages [38].

Our results constitute the first evidence that *Millepora* species produce antimicrobial compounds, whose chemical structure needs to be elucidated. It is very likely that some peptides structurally related to those found in the putative peptidome are responsible for the antimicrobial activity; however, this hypothesis needs to be experimentally confirmed. Antimicrobial compounds synthesized by hydrocorals might represent novel leads for the development of new anti-infective molecules.

### 3.2. Cytolysins from the Nematocyst Proteome of M. complanata

Cnidarian nematocyst venom comprises a complex mixture of toxic proteins and peptides, which are employed for prey paralysis or predator deterrence [39,40]. Similar to what was found in the *Hydra magnipapillata* proteome, the nematocyst proteome of *M. complanata* contained proteins with a molecular mass range between 10 and 70 kDa [31]. However, prominent high molecular mass bands of approximately 200 kDa were observed in the *M. complanata* proteome (Figure 3A). It is estimated that cnidarian venom possesses a high variety of compounds (about 250), mainly of proteinaceous origin [41]. Here, 575 protein spots were detected in the nematocyst proteome of *M. companata* (Figure 3B). These findings could imply that hydrocorals synthesize a greater structural diversity of toxins compared to other cnidarians such as *Cyanea capillata* (53 putative toxins)*, Nemopilema nomurai* (69 putative toxins) [42], *Chrysaora fuscescens* (163 proteins) [43], *Cyanea nozakii* (20 proteins)*, Chrysaora caliparea* (12 proteins), and *Lychnorhiza malayensis* (8 proteins) [44].

Although differences in the venom composition of cnidarians belonging to different classes have been observed, cytolytic toxins are very important components of cnidarian venoms [45]. In fact, cytolysis is a well-known effect induced by toxins of cnidarians, including hydrozoans, such as *Physalia physalis*, *Pandea rubra*, *Arctapodema* sp., and *Colobonema sericeum*, among others [46]. Regarding *Millepora* species, potent hemolytic activity has been reported for *M. complanata* [47], *M. alcicornis* [24], *M. dichotoma* [29], and *M. platyphylla* [30]. The zymographic analysis of the nematocyst proteome of *M. complanata* showed two zones of hemolytic activity that were detected at ~25 and ~205 kDa (Figure 4A). These results were in accordance with a previous study carried out by our research group on *M. alcicornis*, which indicated that the venom of this hydrocoral contains hemolysins with molecular mass of ~28–30 kDa (with PLA2 activity) and ~200 kDa [24]. These findings suggest that both hydrocorals synthesize hemolytic proteins with similar molecular mass. The PLA2 zymogram revealed two zymolytic bands at approximately 6 and 25 kDa (Figure 4B). Low molecular mass toxic PLA2s have been found in some cnidarians, including *Adamsia carciniopados* (13.5 kDa) [8], *Bunodosoma caissarum* (14.7 kDa) [48], *Condylactis gigantea* (14.5 kDa) [49], and *Urticina crassicornis* (12.4 kDa) [50]. However, the PLA2s found in these cnidarians had molecular masses greater than 12 kDa. Thus, the presence of a 6 kDa PLA2 in the proteome of *M. complanata* nematocysts could indicate that this hydrocoral synthesizes PLA2s with structural characteristics different from those of other cnidarian PLA2s, whose structural features remain to be elucidated.

Furthermore, the zymograms of proteolytic enzymes showed bands within a molecular mass range of 50 to 205 kDa (Figure 4C). Proteases with approximate molecular mass of 30 kDa have been described as important constituents of some jellyfish venoms [51]. Particularly, it has been found that proteases of the astacin-like metalloprotease family are ubiquitous components of cnidarian venoms. A 32.3 kDa homolog of astacin-like metalloproteinase was detected in the hydrozoan *Podocoryne carnea* [52]. Another astacin-like toxin of 30.38 kDa has been identified in the venom of *Stomolophus meleagris* [51]. Nevertheless, proteases higher than 50 kDa have not been previously reported for any *Millepora* species. The high molecular mass metalloproteases found in the venom of *M. complanata* could play an important role in capturing prey and digestion.

The results obtained through zymography agree with those derived from the transcriptome analysis, which suggested that the nematocyst proteome of *M. complanata* contains a mixture of hemolysins, proteases, and phospholipases. The presence of various types of toxins in the venom of *M. complanata* indicate that this organism possesses a sophisticated biochemical system involved in efficient capture and digestion of prey. To further characterize the hemolytic activity elicited by the *M. complanata* nematocyst proteome, we assessed the influence of specific enzymatic inhibitors. EDTA was capable of reducing 44.2% of the hemolysis induced by the hydrocoral nematocyst proteome, which implies that almost half of the cytolysins responsible for this activity are divalent ion-dependent toxins. In fact, divalent ions such as Ca^2+^ are necessary for conformational changes in enzyme catalysis and toxin activity of cnidarian proteases [53]. Moreover, inhibition of the hemolytic effect by trypsin, chymotrypsin, serine-proteases, and matrix metalloproteases inhibitors indicated the involvement of several proteases in the hemolytic activity elicited by the proteome of *M. complanata* nematocysts. Considering that, in a previous study, our research group identified the astacin-like metalloprotease toxin 5 in the soluble proteome of *M. complanata* [26], it is very likely that some of the proteases responsible for the hemolytic effect induced by the proteome of *M. complanata* nematocysts belong to the astacin family of metalloproteases. Several studies have shown that these types of toxins are common components of cnidarian venoms [54,55,56], where they act as spreading agents, which facilitate the diffusion of other toxins contained in the same venom.

Although the mechanism of hemolysis induced by cnidarian venoms has not been completely characterized, it has been previously demonstrated that, in the case of *Millepora* species, phospholipases A2 significantly contributes to hemolytic activity. In the present study, we found that the PLA2 inhibitor Varespladib significantly diminished (19.66%) the hemolytic activity of the *M. complanata* nematocyst proteome, confirming that this effect can be partly attributed to the presence of PLA2s.

## 4. Conclusions

In this study, we determined the toxin diversity of the “fire coral” *M. complanata* using a transcriptome data mining approach. Metalloproteases, hemostasis-impairing toxins, and phospholipases (including phospholipases A and B) were predominant putative components of this “fire coral” venom. The hemolytic activity elicited by the nematocyst proteome of this hydrocoral can be ascribed to the presence of toxic proteases (including trypsin, chymotrypsin, serine-proteases, and metalloproteases) and PLA2s. On the other hand, the prediction of antimicrobial peptides revealed homologs to SK84, cgUbiquitin, Ubiquicidin, TroTbeta4, SPINK9-v1, and Histone-related antimicrobials. Moreover, the *M. complanata* nematocyst peptidome exhibited broad-spectrum antimicrobial activity against both Gram-negative and Gram-positive bacteria including *S. enteritidis, P. perfectomarina, E. coli*, and *C. xerosis*. This is the first report regarding the toxin diversity and antibacterial activity of the venoms produced by *Millepora* species, which will contribute to broadening our understanding of the mechanisms underlying the toxic effects produced by these important cnidarians. Additionally, the potential of these hydrocorals as a valuable source of structurally and functionally diverse biomolecules was evidenced.

## 5. Materials and Methods

### 5.1. Ethics Statement

All experimental procedures described in this study were carried out after obtaining authorization by the ethics committee of the Faculty of Chemistry of the Autonomous University of Queretaro (Permit number CQB19/058). *M. complanata* specimen sampling was authorized by the Mexican Ministry of Environment and Natural Resources-SEMARNAT (Permit number PFP/DGOPA-139/15).

### 5.2. Transcriptomic Data Acquisition

The *M. complanata* assembled transcriptome was downloaded from the NCBI Transcriptome Shotgun Assembly (TSA) project (DDBJ/EMBL/GenBank under the accession GIXC00000000.1) (accessed on 1 May 2021) [57].

### 5.3. Functional Annotation and Gene Ontology

Sequences from the transcriptome were annotated using sequence homology against the UniProtKB/Swiss-Prot database (https://www.uniprot.org/, accessed on 1 June 2021) with the DIAMOND software (e-value threshold of 1.0 × 10^−6^) [58]. GO terms were assigned for Blastx hits using the Blast2Go software [59]. Briefly, sequences were analyzed employing Mapping and Annotation, and GO terms were grouped into the MF domain. GO terms across MF categories were visualized with Python scripts (code repository: https://github.com/vhelizarraga/Fire_coral_analysis.git, accessed on 1 June 2021).

### 5.4. Identification of Putative toxins

*M. complanata* proteome was predicted from transcriptome sequences for all three forward and reverse frames using the Seqinr software [60] (Charif and Lobry, 2007). Thereafter, putative toxins were identified in the predicted proteome using Blastp [61]. Shortly, protein sequences were annotated by sequence homology against the Animal Toxin Annotation Project database version UniProtKB 2021_01 (https://www.uniprot.org/program/Toxins, accessed on 1 July 2021) using an e-value threshold of 1.0E-6. After annotation, the hits were filtered and classified; only sequences with an e-value equal to or smaller than 1.0E-20 and matching a length > 10 amino acids were considered toxins.

### 5.5. Multiple Sequence Alignments

Representative putative toxins (one hemolysin and one PFT) were selected for multiple sequence alignment analysis against the related homolog sequences from the UniProtKB/Swiss-Prot protein database. ClustalOmega was used for sequence alignment (https://www.ebi.ac.uk/Tools/msa/clustalo/, accessed 1 August 2021) [62] and the results were visualized with the Jalview software version 2.11.1.4 [63].

### 5.6. Prediction of AMPs

Sequences with a length smaller than 100 amino acids were retrieved from the predicted *M. complanata* proteome using Linux (code repository: https://github.com/vhelizarraga/Fire_coral_analysis.git, accessed on 2 September 2021). Potential AMPs were identified from these sequences with amPEPpy [64]. The antimicrobial activity was inferred by implementing a random forest classifier using the distribution descriptor set from the Global Protein Sequence Descriptors. Sequences less than 10 amino acids long and containing nonstandard amino acids were not evaluated. Sequences with a predicted probability higher than 0.8 were recovered. Annotations were carried out employing the DIAMOND software [58] against the following two databases: the Data Repository of Antimicrobial Peptides—DRAMP (http://dramp.cpu-bioinfor.org/, accessed on 30 September 2021) [65]; and the Antimicrobial peptide Database—AP3 (https://wangapd3.com/main.php, accessed on 30 September 2021) [66].

### 5.7. Sample Collection and M. complanata Nematocysts Proteome Preparation

Specimens of *M. complanata* were obtained from the Parque Nacional Arrecife de Puerto Morelos, Quintana Roo, Mexico in November 2016. Samples were collected using a chisel and hammer at 4 m depth and stored in liquid nitrogen. Extraction of nematocysts content was carried out by osmotic shock as previously described [24]. This procedure is commonly employed for extracting cnidarians toxins and the resulting extract is enriched with soluble peptides and mainly, but not exclusively, from nematocyst origin [67,68]. Briefly, 200 g of hydrocoral sample was placed in a 500 mL beaker and 300 mL of triple distilled deionized water at pH 7 was added. Protein extraction was carried out employing a seesaw shaker for 24 h at 4 °C. The resulting aqueous extract was centrifuged at 12,000 rpm until sediments were not present. Thereafter, the solution was filtered using a Waltman 40 cellulose filter and frozen at −70 °C. Subsequently, the frozen aqueous extract was lyophilized and the protein content was determined using a 2D Quant kit from GE Healthcare as per the manufacturer’s instructions.

### 5.8. One-Dimensional and Two-Dimensional Polyacrylamide Gel Electrophoresis

The nematocyst proteome was analyzed employing sodium dodecyl sulfate polyacrylamide gel electrophoresis (SDS-PAGE) as previously described [27]. The lyophilized powder containing 80 μg of protein was resuspended in proteomic grade water. Samples (*n* = 3) were resolved in 18% polyacrylamide using Tris-glycine as a running buffer (running conditions: 120 V for 1.5 h). In addition, the nematocyst proteome was analyzed by high-resolution two-dimensional electrophoresis (2DE) as previously described [26]. Samples (750 μg of protein; *n* = 3) were solubilized in rehydration buffer (8M urea, 2% SDS, 0.375M Tris-HCl (pH 8.8), 20% glycerol, and 2% DTT (*w/v*)) and separated by isoelectrofocusing. Immobilized pH gradient strips (pH range 3–10) were loaded on an isoelectrofocusig system Bio-Rad PROTEAN^®^i12™ (Hercules, CA, USA, Bio-Rad) for a total of 20,000 Vh. After isoelectrofocusing, the IPG strips were reduced using equilibration buffer containing 6M urea, 2% SDS, 0.05 M Tris–HCl, pH 8.8, 50% glycerol, and 2% (*w/v*) dithiothreitol (DTT), and alkylated with 6 M urea, 2% SDS, 0.05 M Tris–HCl, pH 8.8, 50% glycerol, and 2.5% (*w/v*) iodoacetamide. Thereafter, samples were run on TGXTM 18% polyacrylamide precast SDS-PAGE gels (CA, USA, Bio-Rad) at 150 V for 2 h at 4 °C. Protein bands and spots were visualized by silver staining. The Precision Plus Protein Dual Color Standards (Bio-Rad, CA, USA) were used for molecular mass estimation.

### 5.9. Zymographic Analysis

Hemolytic and PLA2 activities were assessed using indirect zymography. All zymographic analyses were carried out employing 100 μg of protein. Samples were resolved by SDS-PAGE as previously described. After running SDS-PAGE, gels were washed with 100 mM Tris–HCl for 1 h and incubated with 50 mM Tris–HCl, 140 mM NaCl, 2.5 mM CaCl_2_, pH 7.4 on substratum gels. In the case of hemolytic activity, the gels were incubated for 4 h at 37 °C on substratum gels (agar 1.5%), enriched with washed human erythrocytes (3%). For PLA2 activity detection, gels were additionally washed with 100 mM Tris–HCl and 1.0%, Triton X-100 (pH 7.4) for 1 h to remove traces of SDS and incubated for 15 h at 37 °C on an agarose gel (2%) prepared with 50 mM Tris–HCl and 6% egg yolk. PLA2 from honeybee venom (*Apis mellifera*) was employed as a positive control. On the other hand, the protease activity was determined by direct zymography. Then, 12% SDS-PAGE gels containing gelatin (2 mg/mL) or casein (2 mg/mL) were used for protein separation. Afterward, gels were washed twice for 20 min with Triton X-100 2.5% and then were incubated for 17 h at 37 °C in 0.5 mM CaCl_2_, 20 mM Tris-HCl (pH 7.4). Next, gels were stained with Coomassie Blue R-250 (0.125%) in methanol (45%) and acetic acid (10%) for 1 h. Finally, gels were cleared with ethanol (40%) and acetic acid (10%). Protein bands displaying enzymatic activity appeared as clear bands in the zymogram gels. Zymographic analyses were determined in three biological replicates.

### 5.10. Hemolytic Activity Assay

The hemolytic activity of the *M. complanata* nematocysts proteome was determined as previously reported [24]. In short, samples of human erythrocytes (1%) were suspended in 1 mL Alsever’s solution (120 mM D-glucose, 30 mM sodium citrate, 7 mM NaCl, and 2 mM citric acid, pH 7.4). The following protein concentrations were assessed: 2.18, 3.16, 4.5, 6.6, 7.9, 9.56, 11.48, and 24.54 μg of protein per mL. Samples were incubated at 37 °C for 30 min and centrifuged at 2300 rpm. Subsequently, the free hemoglobin in the supernatant was measured spectrophotometrically at 415 nm. The HU50 was determined using the DRC package of R statistical software (code repository: https://github.com/vhelizarraga/Fire_coral_analysis.git, accessed on 2 October 2021) [69]. Specific inhibitors of hemolysis were used as controls: Nα-Tosyl-L-lysine chloromethyl ketone hydrochloride (TLCK, trypsin inhibitor), N-p-Tosyl-L-phenylalanine chloromethyl ketone (TPCK, chymotrypsin irreversible inhibitor), BB-94; (2R,3S)-N4-Hydroxy-N1-[(1S)-2-(methylamino)-2-oxo-1-(phenylmethyl)ethyl]-2-(2-methylpropyl)-3-[(2-thienylthio)methyl] butanediamine (Batimastat, matrix metalloprotease inhibitor), Phenylmethylsulfonyl fluoride (PMSF, serine protease inhibitor), Ethylenediaminetetraacetic acid (EDTA, non-selective protease inhibitor), and 2-[[3-(2-Amino-2-oxoacetyl)-2-ethyl-1-(phenylmethyl)-1H-indol-4-yl]oxy]-acetic acid (Veraspladib, sPLA2 inhibitor). The optimum concentration of each inhibitor was standardized according to previously reported concentrations. Inhibition induced by each inhibitor on the hemolytic activity was measured after incubation for 30 min at 37 °C.

### 5.11. Antibacterial Activity Assay

The nematocysts proteome of *M. complanata* was filtered using Amicon^®^ filter tubes according to the manufacturer’s instructions (Amicon^®^ Pro Purification System, Merck KGaA, Darmstadt, Germany). Then, the antibacterial activity of this proteome fraction, containing peptides smaller than 10 kDa, was evaluated against 17 species of Gram-positive and Gram-negative bacteria by the disc diffusion method. The following bacteria strains were employed: *Salmonella agona*, *Salmonella typhimurium*, *Salmonella enteritidis*, *Salmonella infantis*, *Salmonella typhi*, *Pseudomonas aeruginosa*, *Pseudomonas perfectomarina*, *Escherichia coli*, *Corynebacterium xerosis*, *Kytococcus dedenturius*, *Exiguobacterium amuntiacum*, *Bacillus koreensis*, *Micrococcus luteus*, *Microbacterium oleivorans*, *Staphylococcus cohnii*, *Staphylococcus xylosus*, and *Staphylococcus aureus*. In short, plates containing Muller-Hinton agar were inoculated with 10 μL of bacterial suspension (adjusted to the McFarland standard No.1). Plates were dried for 15 min and then employed for the sensitivity test. Sterile discs were impregnated with *M. complanata* peptidome samples (40 μg protein/disc; *n* = 3) and placed on the Muller–Hinton agar plates. Then, plates were incubated at 37 °C for 24 h. Inhibition zone diameters were determined after incubation. The MIC was assessed by the agar microdilution method using 96-well plates. MIC was determined employing the following protein concentrations: 400, 40, 4, 0.4, 0.04 and 0.004 µg/µL. All analyses were carried out in triplicate using bacterial suspensions adjusted to the McFarland standard No. 1. The 96 well plates were incubated at 37 °C for 24 h and the MIC was calculated spectrophotometrically at 600 nm.

## Figures and Tables

**Table 1:** Top ten antimicrobial peptides (AMPs) hits from the *M. complanata* predicted peptidome.

SeqID	Accession	Description	Activity	E-Value	Sequence
Mc1655639	DRAMP03113	SK84	Anti-Gram+; antifungal; antiviral	1.3 × 10^−9^	LPSFSGSMFNPEGMPGSGAGKGGGGGGSVRDAGGSFGKMEAAREEEYFRRLQKEQLKSLQHQLDEEVDHHERELKQ
Mc2434582	DRAMP03472	cgUbiquitin	Anti-Gram+; anti-Gram−; antifungal	1.5 × 10^−37^	GGMQIFVKTLTGKTITLEVEPSDTIENVKAKIQDKEGIPPDQQRLIFAGKQLEDGRTLSDYNIQKESTLHLVLRLRGGMQIFVKTLTGKTITLEVEP
Mc1269566	AP02096	Ubiquicidin	Anti-Gram+; anti-Gram−; anti-MRSA	6.1 × 10^−23^	GIPNLSTINVVAQVLGGKVHGSLARAGKVKGQTPKVDKQDKKKKKTGGSHRRIQYNRRFVNVVPSFGRRRGPNSNNNS
Mc2063087	AP02804	Histone H2A	Anti-Gram+	2.6 × 10^−17^	RHLKNRTTSHGRVGATAAVYSAAILEYLTAEVLELAGNASKDLKVKRITPRHLQLAIRGDEELDAL
Mc1970206	AP02807	Histone H4	Anti-Gram−	8.6 × 10^−23^	ETRGVLKVFLENVIRDAVTYTEHARRKTVTAMDVLYARKRQGKTLYGFGGGWGSLEWGEVAVGSPRDQAETNPRMLVG
Mc2019202	AP02808	Histone H2B	Anti-Gram−	1.7 × 10^-12^	SREIQTAVRLILPGELAKHAVSEGTKAVKKYNSMAELYIIIIKIKQFFFFFFFFFFFSSFFFSIFLYSIKP
Mc1808465	AP02809	Histone H3	Anti-Gram−	3.3 × 10^−36^	RKLPFQRLVREIAQDFKTDLRFQSTAVMALQEASEAYLVGLFEDTNLCAIHAKRVTIMPKDIQLARRIRGERATLQTKNGYFYS
Mc1970206	AP02810	Histone H4	Anti-Gram−	5.1 × 10^−23^	ETRGVLKVFLENVIRDAVTYTEHARRKTVTAMDVLYARKRQGKTLYGFGG*GWGSLEWGEVAVGSPRDQAETNPRMLVG
Mc1777530	AP02896	TroTbeta4	Anti-Gram+; anti-Gram−	2.8 × 10^−11^	RIIRSAFREVFSCFLFIHQSVVMGDKPDVSGVTTFDKSKLKKAETQEKNTLPTKETIEQEKSGDVKLVCTISTRPLLQFYSV
Mc1329197	AP03038	SPINK9-v1	Anti-Gram−, enzyme inhibitor	9.5 × 10^−7^	CKFDKKTCKSSCVLLSKYKCNDKCLDIYKPVCGSDGRTYSNQCELDLASCKSNGKIKKVSDGECTNAVLHQILVNIVAFHTPRQNI

**Table 2:** Susceptibility of bacterial strains to the effect of *M. complanata* nematocysts peptidome.

	Species	Antibacterial Activity	Zone Of Inhibition (mm)	MIC ^a^ (µg/mL)
Gram(−)	*Salmonella agona*	−	NA	NA
	*Salmonella typhimurium*	+	20 ± 1	0.4
	*Salmonella enteritidis*	+	18 ± 0.5	0.04
	*Salmonella infantis*	+	22 ± 1	4
	*Salmonella typhi*	+	20 ± 1	>4
	*Pseudomonas aeruginosa*	+	12 ± 0.5	4
	*Pseudomonas perfectomarina*	+	20 ± 1.5	0.04
	*Escherichia coli*	+	22 ± 1	0.04
Gram(+)	*Corynebacterium xerosis*	+	12 ± 0.5	0.04
	*Kytococcus dedenturius*	−	NA	NA
	*Exiguobacterium amuntiacum*	−	NA	NA
	*Bacillus koreensis*	+	20 ± 0.5	4
	*Micrococcus luteus*	−	NA	NA
	*Microbacterium oleivorans*	+	17 ± 0.5	4
	*Staphylococcus cohnii*	−	NA	NA
	*Staphylococcus xylosus*	−	NA	NA
	*Staphylococcus aureus*	−	NA	NA

^a^ Minimum inhibitory concentration, (+) Active, (−) No active, (NA) Not assessed.

## Data Availability

The data presented in this study are available in https://github.com/vhelizarraga/Fire_coral_analysis.git (accessed on 2 October 2021).
